# Pluripotency and its layers of complexity

**DOI:** 10.1186/2045-9769-1-7

**Published:** 2012-09-14

**Authors:** Jolene Ooi, Pentao Liu

**Affiliations:** 1_6 Wellcome Trust Sanger Institute, Hinxton, CB10 1SA UK; 2_6 Technology and Research, Agency for Science, 1 Fusionopolis Way, #20-10, Connexis North Tower, Kragujevac, 138632 Singapore

**Keywords:** Naïve pluripotency, Primed pluripotency, Embryonic stem cells, Induced pluripotent stem cells, Epiblast stem cells

## Abstract

Pluripotency is depicted by a self-renewing state that can competently differentiate to form the three germ layers. Different stages of early murine development can be captured on a petri dish, delineating a spectrum of pluripotent states, ranging from embryonic stem cells, embryonic germ cells to epiblast stem cells. Anomalous cell populations displaying signs of pluripotency have also been uncovered, from the isolation of embryonic carcinoma cells to the derivation of induced pluripotent stem cells. Gaining insight into the molecular circuitry within these cell types enlightens us about the significance and contribution of each stage, hence deepening our understanding of vertebrate development. In this review, we aim to describe experimental milestones that led to the understanding of embryonic development and the conception of pluripotency. We also discuss attempts at exploring the realm of pluripotency with the identification of pluripotent stem cells within mouse teratocarcinomas and embryos, and the generation of pluripotent cells through nuclear reprogramming. In conclusion, we illustrate pluripotent cells derived from other organisms, including human derivatives, and describe current paradigms in the comprehension of human pluripotency.

## Review

### Introduction

Pluripotency is denoted by the capacity of a self-renewing cell to develop into the three germ layers. The conception of pluripotency emerged in the Classical Greek period where rudimentary methods were employed to examine the development of organs within chick embryos [1]. These observations were left unexplored for two thousand years and awareness was rekindled in the Renaissance period where the invention of the microscope enhanced the resolution of developing embryos. This elicited the establishment of several landmark discoveries and escalated our understanding of vertebrate developmental processes.

The first testimony of pluripotency on a petri dish was portrayed using inbred strains of mice. Spontaneous incidences of teratocarinomas arose at low frequencies in 129 strain of mice [2]. This led to the isolation of pluripotent stem cells that were able to regenerate tumours consisting of the three germ layers [3]. Subsets of pluripotent cell populations came in quick succession, where various cell types within the mouse embryo were isolated and snapshots of distinct developmental stages were captured [4–9]. Other than naturally occurring instances in normal development, artificial states that are reflective of pluripotency have been accomplished. Cells from developing *Rana pipiens* embryos were demonstrated to undergo nuclear transplantation and revert to a primitive state capable of developing into an entire organism [10]. This highlighted the capacity of a non-pluripotent cell to reset its epigenetic marks and convert to a pluripotent derivative. Termed as nuclear reprogramming, these findings were extended in mice and further exemplified in alternative methods [11–14].

The easy manipulation and cultivation of mouse pluripotent stem cells have provided a convenient platform to study the independent developmental stages. Furthermore, comparison of these pluripotent states and their necessary environmental milieu for sustenance provides indications of developmental cues (reviewed by [15]). Pluripotent stem cells from various non-rodent and primate species have been achieved either directly from embryos or through nuclear reprogramming, but none are truly reflective of mouse embryonic stem cells that display germline competence (reviewed by Nichols and Smith, 2009c) [16]. Recent studies suggest that conventional human pluripotent cells resemble mouse epiblast stem cells more closely than mouse embryonic stem cells [9], indicating the possibility of a primitive subset of human pluripotent stem cells which have not been clearly delineated.

This review aims to address these concerns by first describing the milestones established through the study of vertebrate development and pluripotency. This will be followed by the illustration of extrinsic signals and molecular pathways associated to pluripotency. By way of introducing pluripotent stem cells achieved from alternative organisms, we compare the differences between human and mouse pluripotent stem cells and describe recent inferences on a distinct state of human pluripotency.

#### History of vertebrate development

The development of vertebrates involves the orchestration of a series of steps in a tightly regulated process that determines cell lineage specification into endodermal, ectodermal and mesodermal derivatives. Imprinted into the operational dogma of modern developmental biology, conception of these notions has been accompanied by a history of key observations and controversies.

Originating from examinations of the chick embryo, Aristotle witnessed the development of a palpitating heart, head and eyes, laying ink on a clean palette of embryology [1]. With the proposition of epigenesis, he described development as a sequential process involving the formation of organs to construct a complete organism. Almost two thousand years after these initial recordings, the field was reawakened and the mechanisms behind these phenomena were questioned. To examine the root of development, Girolamo Fibrici performed dissections on cadavers of pregnant mammals, providing comparisons between anatomical structures of uteri [17]. This work was advanced by his student William Harvey who hypothesised the presence of female germ cells within uteri that hold the capacity to constitute a new organism ([18]). Furthermore, identification of budding and subdivision during primary stages of embryonic development of the chick led him to be a strong advocate of epigenesis. These findings revived Aristotle’s theory and provoked collision against preformation views. Preformationism was held as the dominant perception of development, and describes the existence of a miniature organism that expands without increasing complexity within the germ cell. Although epigenesis perceptions were resurrected, it was not received warmly. Transformation of the field of development biology was invoked by subsequent experiments led by Caspar Frederich Wolff and Karl Ernst von Baer. Using plants as a surrogate organism for study, Wolff explained the ability of differentiated plant root to regenerate a new organism. This study was traversed to chick embryos where Wolff studied the formation of embryonic kidneys [19]. Building on the scaffold of information uncovered by his predecessors, von Baer discovered the presence of primitive germ cells and ultimately eclipsed any influence of preformationism [20].

#### Beginnings of the notion of pluripotency

Delineating vertebrate developmental processes proposes the presence of pluripotent cells which participate in the contribution of the cellular entirety of an adult organism. The first inkling of pluripotency was established by Hans Driesch who demonstrated that isolation of individual cells within 2-cell embryos culminated in the generation of two small complete larvae [21]. In parallel, Hans Spemann validated the ability of detached cells within 2-cell newt embryos to develop into intact organisms [22]. As Spemann was poised with micro-surgical skills, he extended his findings in embryology through the constriction of developing embryos using baby hair. By restricting the position of the nucleus to one side of the cytoplasm, development of the embryo into its 16-cell state would result in the escape of one cell past the noose, into the opposite end. This led to the formation of twin larvae, suggesting the pluripotent capacity of cells within the developing embryo. These findings illuminated a new era of embryological study and unravelled avenues for the study of pluripotency.

#### Embryonic carcinoma cells

To reinforce the notion of pluripotency, pluripotent cells have been successfully established on the Petri dish. Teratocarcinomas are tumours discovered in humans and mice ([2, 23]. Inspection of these cellular masses reveals the presence of a plethora of organised structures, including teeth, fingers and hair, suggesting the presence of pluripotent cells within the tumour. This was corroborated through the determination that intraperitoneal injection of a single cell could generate teratocarcinoma consisting of an array of differentiated tissues [3]. Likewise, grafting of mouse embryos into adult mice also leads to the formation of teratomas, reinforcing the existence of all-encompassing cells ([24, 25]). Teratomas in both contexts have been successfully maintained in culture [26–29]. Designated as embryonic carcinoma (EC) cells, these cells exhibit pluripotent properties including the ability to form teratomas in immune-compromised mice and serve as the first platform to study embryonic development of mice *in vitro*.

#### Embryonic stem cells

Soon after, the inner cell mass of mouse blastocysts was demonstrated to be sustained on a petri dish, recapitulating an early developmental event *in vitro*[5, 6]. Labelled as embryonic stem (ES) cells, these cells were competent at contributing to the three germ layers in teratomas, when injected into immune-compromised or syngenic mice. Furthermore, re-introduction of these cells into the mouse blastocyst led to the formation of high percentage chimeras, indicating their ability to participate in normal murine development, a property not frequently shared with EC cells. To satisfy stringent pluripotent stipulations, ES cells were also studied for their ability to contribute to the germline and an intact embryo. The former was approached through the cross of chimeras to phenotypically distinct wildtype mice [30], whereas the latter was addressed through tetraploid complementation assays [31, 32].

Amenable to modifications, ES cells embody a useful tool for genetic alterations (reviewed in [33]). With the elucidation of the genetic composition of the mouse in 2002 [34], the genomic content of ES cells has been frequently disrupted in a precise fashion to study gene function. The capacity for germline transmission results in the establishment of intact mice harbouring any desired genetic mutation in the germline [35–38].

#### Epiblast stem cells

ES cells represent a subset of cells isolated from the epiblast in pre-implantation blastocysts and depict a primitive developmental stage of the developing embryo. To recapitulate late phases, two independent groups have segregated the columnar epithelial epiblast of the early post-implantation embryo and cultivated it on a petri dish [4, 9]. Termed as Epiblast stem cells (EpiSCs), they behave distinctly from ES cells and are rarely able to generate chimeras. However, both ES cells and EpiSCs are competent in multi-lineage differentiation, where injection of these cells into immune-compromised mice results in the development of teratomas comprised of tissue types characteristic of the three germ layers [4, 9].

Examination of the molecular circuitry within these cells revealed some similarities to ES cells, where the core transcriptional machinery consisting of Oct4, Sox2 and Nanog was expressed [39–41]. However, ES cells and EpiSCs exhibit disparities in transcript and epigenetic levels of markers associated to the inner cell mass and early germ layers [9], highlighting distinctions in their original developmental stages.

#### Embryonic germ cells

Pluripotent stem cells divergent from the mouse blastocyst were first derived from primordial germ cells (PGCs) [7, 8]. Emergent at 7 days post coitum (dpc), these cells are represented by a small population of alkaline-phosphatase positive cells [42]. In a span of six days, these cells undergo extensive proliferation every 16 hours to comprise of 25,000 PGCs [43], and eventually reside in either the testis or ovary of the mouse.

Extraction of PGCs at 8.5-12.5 dpc from the posterior fragment of the embryo and cultivation in the presence of soluble factors such as leukemia inhibitory factor (LIF), steel factor (SF) and fibroblast growth factor (FGF), results in a population of cells that exhibits self-renewal and limitless proliferation ([7, 8, 44, 45]). Coined as embryonic germ (EG) cells, these cells resemble ES cells and are capable of generating chimeras and contributing to the mouse germline [7, 46, 47].

#### Artificial states of pluripotency

Historical perspectives on the study of vertebrate development illuminated the remarkable capacity of a fertilized oocyte to generate a complete organism. This led to the advent of nuclear reprogramming, denoted by the transition between unrelated cell types triggered by switches in gene patterns. Introduction of a somatic cell nucleus into an enucleated oocyte results in the re-establishment of epigenetic marks, allowing the hybrid cell to generate an organism ([10]; [48, 49]). Termed as Somatic Cell Nuclear Transfer (SCNT), this technique has proved effective across species [11, 50], but is subject to technical competence and coordinated mitotic cycles (reviewed by [51]).

A similar phenomenon is observed upon the fusion of pluripotent stem cells and somatic cells. The environmental milieu arising from the pluripotent cell seizes control of the cellular transcriptional machinery and leads to the silencing of somatic markers in the fusion cells. The resultant heterokaryon is tetraploid but able to differentiate into all three germ layers [14, 52].

Recently, ectopic expression of four transcription factors was described to revert somatic cells to a pluripotent state [13]. For the ease of nomenclature, these resultant cells were termed as induced pluripotent stem (iPS) cells. Mouse iPS cells are similar to mouse ES cells, and are capable of generating chimeras and contributing to the germline [53]. Traversed across species, human iPS cells have since been obtained [54, 55]. The ability to generate patient specific iPS cells highlights its potential for cell therapy, drug screening and disease modelling (reviewed by [56]). Comprehensive studies of iPS cells and ES cells reveal minute differences in phosphoproteomic and transcriptomic components that were statistically disregarded [57]. However, epigenomic analysis at high resolution expose subtle differences between iPS cells and ES cells [58, 59] and continuous in vitro culture could incur genomic aberrations [58, 60, 61]. These discrepancies could account for functional disparities such as epigenetic memory [62–64] and immunogenicity [65], surmising potential for improvement in the derivation of iPS cells which possess qualities that are identical to ES cells.

#### Chemicals and pathways associated with murine pluripotent stem cells

Pluripotent cells exist in a fleeting manner within the mouse embryo, placing emphasis on the remarkable extension of their life in culture. Supporting chemicals or matrix are necessary for the maintenance of pluripotency in culture, as exemplified in the reliance on fibroblasts and serum in primary studies describing ES cells, EC cells and EG cells, suggesting a non-cell autonomous mechanism in self-renewal. Extrication of components that support pluripotency, in concert with our current understanding of developmental pathways, can lead to the improvement of growth parameters of pluripotent cells, and augment our knowledge on embryonic development.

Stemming from the discovery that medium conditioned by Buffalo rat liver cells was sufficient to retain pluripotency [66], the active component necessary for this phenomenon was narrowed down to leukemia inhibitory factor (LIF) [67, 68]. The importance of LIF has been portrayed by its obligatory need in the culture medium [69], acting through gp130 and the recruitment of JAK kinase and STAT3 [70, 71]. The ability of LIF to maintain pluripotency in culture is mirrored in a physiological context, where LIF and gp130 are expressed in early embryos and during diapause [72].

Although LIF/gp130 and their related pathways are pivotal in the maintenance of pluripotency, the use of chemically defined basal media supplemented with N2, B27 and LIF is unable to impede differentiation of ES cells into neuronal derivatives [73]. This propensity to differentiate can be restrained by the addition of bone morphogenetic protein (BMP), an anti-neural factor in vertebrate development [74]. Functioning through Inhibitor of differentiation (Id), BMP together with LIF are sufficient to drive ES cells into self-renewal without differentiation [73].

Interaction between LIF and gp130 triggers a conflicting response, where both the JAK/STAT and ERK1/2 pathways are activated [75, 76]. As the latter stimulates differentiation, ERK or FGF inhibitors were demonstrated to circumvent this impediment and support the maintenance of ES cells [77]. Activation of the Wnt pathway through the inhibition of GSK3β also assists in the sustenance of an undifferentiated state. The effects of ERK and GSK3β inhibition (2i) are compounded when used in combination, and results in a homogenous population of primitive cells designated as ground state pluripotency [78]. Mirroring this *in vivo*, addition of 2i to early mouse embryos in culture causes an expansion of the Nanog-expressing epiblast at the expense of the hypoblast and trophoectoderm compartments [79]. Growth media containing 2i and LIF has also made it possible to derive ES cells from mouse strains, such as CBA and NOD, and rats, which have been recalcitrant to previous methods [77, 80–82].

The culture conditions of EpiSCs are distinct from ES cells. FGF and Activin are necessary to preserve EpiSCs [4, 9], whereas the addition of 2i and LIF generally results in cell differentiation or death [83]. In contrast, addition of an Activin inhibitor led to widespread differentiation, suggesting reliance on Nodal/Activin signalling [9].

The disparities reflected by dissimilar developmental potential and growth conditions of mouse ES cells and EpiSCs has led to the notion of naïve and primed pluripotency (Nichols and Smith, 2009c). Originating from the pre-implantation epiblast, ES cells display complete pluripotent potential and are capable of germline contribution. In contrast, EpiSCs derived from the post-implantation epiblast are incapable of neither somatic nor germline contribution, exhibiting limited pluripotent potential.

Classification of naïve and primed pluripotency has been strengthened by the analysis of X-chromosome inactivation (XCI). XCI is a process where one X chromosome in female diploid cells is inactivated, resulting in the reduction of most X-linked transcripts to comparable levels between males and females ([84]; reviewed by [85]). Apart from EpiSCs, mouse pluripotent cells (ES, EC, EG, iPS cells) display two copies of active X chromosomes [86–88]. This is reminiscent of pre-implantation epiblasts which possess two active X chromosomes and hold the capacity to derive complete organisms, exemplifying naïve pluripotency [89, 90]. In contrast, EpiSCs exhibit XCI [83] or primed pluripotency and represent post-implantation epiblasts where XCI begins and the capacity to form whole organisms is lost.

#### Establishment of ES cells from other species

After successful isolation and culture of mouse ES cells, there have been several attempts at engineering an equivalent in various species, including rodents such as hamsters and rats [80, 81, 91], non-rodents such as rabbits, minks, chickens, pigs and cows [92–98], and primates such as rhesus monkeys and the common marmosets [99, 100].

Heightened interest in the generation of an array of pluripotent stem cells can be attributed to its potential to differentiate into an array of cell types, representative of the three germ layers. Application of this technology to humans illuminates the possibility of regenerative medicine. To address this, human ES cells were derived from cleavage stage human embryos that were acquired from *in vitro* fertilization donors [101].

With the successful establishment of human ES cells, much effort has been directed at understanding the pathways involved in maintaining the pluripotent cells in culture and modifications to growth conditions of human ES cells have since been implemented. Unlike mouse ES cells, LIF signalling is not sufficient to sustain undifferentiated human ES cells [102, 103]. In addition, BMP4 triggers trophectoderm differentiation in human ES cells [104, 105]. Instead, FGF and Nodal/Activin signalling pathways have been implicated in the self-renewal of human ES cells, reminiscent of EpiSCs [106–109]. Furthermore, comparison of gene expression patterns and XCI further supports the resemblance between human ES cells and EpiSCs [110].

Following the inception of transcription factor induced reprogramming, an array of human somatic cell types have been competently reprogrammed to derive human iPS cells [54, 55, 111, 112]. Human iPS cells represent an attractive source of patient-specific pluripotent cells and evade ethical concerns faced by the usage of human ES cells. Cultivated in a similar environment to human ES cells, human iPS cells display close resemblance when protein and epigenetic signatures are compared [57, 58], hence are also likened to EpiSCs.

#### Differences between human and mouse ES cells: naïve and primed pluripotency

The similarities between human pluripotent cells (human ES cells and iPS cells) and primed pluripotent mouse EpiSCs propose the possible existence of an unexplored naïve human pluripotent state reminiscent of mouse ES cells. Using human embryos, it has been recently demonstrated that introduction of inhibitors against FGF, ERK or GSK3 did not reduce the Nanog-expressing epiblast compartment, highlighting a stark contrast to conventional human pluripotent cells which readily differentiate in similar conditions [113, 114]. An additional study which traced the origin of human ES cells derived from human blastocysts noted that the establishment of human ES cell lines required the transition into a post-ICM intermediate displaying X-inactivation ([115]).

Murine EpiSCs can be converted into ES cells with the ectopic expression of Klf2, Klf4, Nr5a2, or the addition of 2i and LIF ([83, 116], [117]). Likewise, human ES cells have been manipulated to phenotypically resemble mouse ES cells [118]. Using iPS cell technology, similar cellular states have been achieved using chemical cocktails or various transgenic combinations [88, 118–120]. In some of these findings, both copies of X chromosomes are active, suggesting the possible attainment of naïve pluripotency. Strikingly, these novel pluripotent states rely on continuous transgenic expression, and only one study established human iPS cells that exhibited XCI reversion and transgene independence [88].

**Figure 1:**
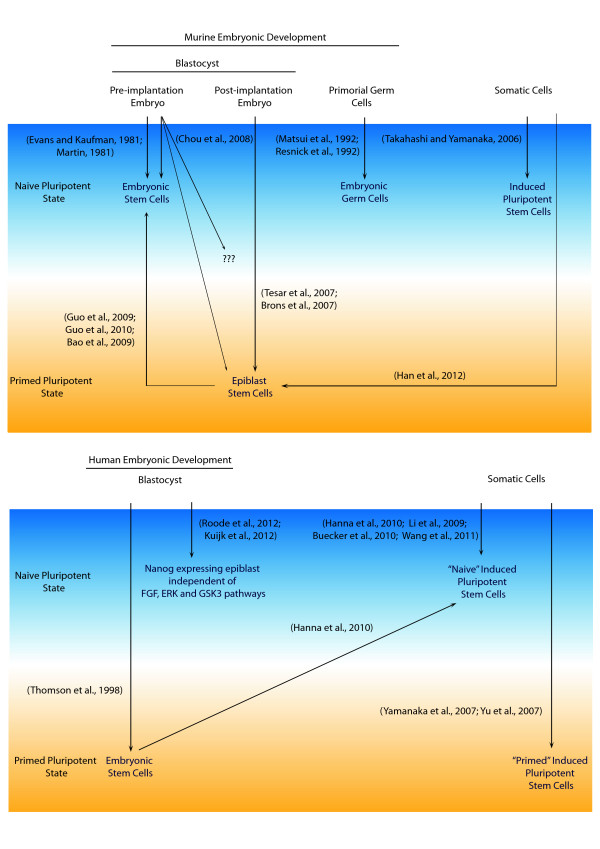
**Different Pluripotent States in Mouse and Human.** (TOP) Distinct pluripotent states derived *in vitro* from developing mouse embryos or through nuclear reprogramming of somatic cells. Further manipulation through the introduction of genetic factors or modification of growth conditions leads to the attainment of additional pluripotent states, contributing to the complexities of pluripotency. (BOTTOM) Pluripotent states captured *in vitro* from human blastocysts or nuclear reprogramming of somatic cells. Modelled after pluripotent states during murine development, recent findings suggest the presence of a naïve pluripotent state during human development.

The study of pluripotency has amassed a wealth of information (Figure 1), from the discovery of nuclear reprogramming in the 1952, to the isolation of mouse ES cells and EpiSCs in 1981 and 2007 respectively. As the first vertebrate model organism used to study pluripotency, our understanding of the various stages during mouse embryonic development has thrived. Translating this knowledge to the developmental processes in other organisms may shed light on species-specific embryological properties, or offer refinements to the strategies employed for the study of development.

The identification of human pluripotent cells and their comparison to murine counterparts is one such example. Disparate chemical components within the growth media plays a critical role in the observed differences between primed human pluripotent cells and naïve mouse ES cells. The generation of mouse ES cells from the inner cell mass of the blastocyst involves the acquisition of new genetic profiles associated to self-renewal, epigenetic regulation and arrest of normal development [121], emphasising the influence of culture conditions on the establishment of cell lines. Furthermore, growth conditions can be manipulated to derive pluripotent cell lines from blastocysts which are distinct from mouse ES cells and EpiSCs [122], and EpiSC-like derivatives from fibroblasts using the conventional cocktail of reprogramming factors [123].

Although conventional human pluripotent stem cells resemble mouse EpiSCs closely, there exist differences between the two. EpiSCs express cell surface marker SSEA-1, whereas conventional human ES cells and iPS cells display SSEA3/4 ([9]; reviewed by [124]). In addition, human ES cells, express pluripotency markers DPPA3, KLF4 and REX1, unlike EpiSCs [39, 110].

As the study of human pluripotency is in its infancy, parallel studies in mouse models can only serve as a guide and may not faithfully recapitulate the physiological events which occur during human development. However, recent pieces of evidence suggest the existence of a naïve state of pluripotency and may surmount difficulties met with FGF-dependent human ES cell and iPS cells. These include susceptibility to harsh dissociation, inability to survive in single cell suspension and genomic instability [125–130]. In conclusion, there remains much to be unearthed for the full elucidation of human embryonic development and further experiments are essential to uncover the proposed subset of naïve pluripotent stem cells.

## Conclusion

This article highlights advancements in the study of pluripotency, evolving from primary embryological experiments, to the recapitulation of an array of pluripotent states *in vitro*. Murine ES cells were first reported in 1981 [5] and represented an amenable and convenient platform to study developmental pathways and the sustenance of pluripotency. The identification of EG cells, EpiSCs and iPS cells led to the comprehension of various developmental stages. It was only in 1998 that success was met with human ES cells [101]. Armed with 17 more years of research, mouse pluripotent states serve as a reference point when delineating human pluripotency. With the advent of iPS cells and sophisticated technical resources, knowledge garnered from the study of human ES cells and iPS cells will continue to amass exponentially, addressing our concerns on naïve pluripotency.

## Abbreviations

ES cells: embryonic stem cells
EC cells: embryonic carcinoma cells
EpiSCs: Epiblast stem cells
EG cells: embryonic germ cells
iPS cells: induced pluripotent stem cells
LIF: leukemia inhibitory factor
2i: two inhibitors
FGF: fibroblast growth factor
XCI: X-chromosome inactivation
BMP: Bone morphogenetic protein
PGC: Primordial germ cell.
